# Relative substituent orientation in the structure of *cis*-3-chloro-1,3-dimethyl-*N*-(4-nitro­phen­yl)-2-oxo­cyclo­pentane-1-carboxamide

**DOI:** 10.1107/S1600536814017711

**Published:** 2014-08-06

**Authors:** Matthias Zeller, Jonas Warneke, Vladimir Azov

**Affiliations:** a Department of Chemistry, Youngstown State University, 1 University Plaza, Youngstown, Ohio 44555, USA; b University of Bremen, Department of Chemistry, Leobener Str. NW 2C, D-28359 Bremen, Germany

**Keywords:** crystal structure, hydrogen bonds, π–π stacking, methacryloyl chloride dimer, Diels–Alder reaction

## Abstract

The structure of the title compound, C_14_H_15_ClN_2_O_4_, prepared by reaction of a methacryloyl dimer with nitro­aniline, was determined to establish the relative substituent orientation on the cyclo­penta­none ring. In agreement with an earlier proposed reaction mechanism, the amide group and the methyl group adjacent to the chloro substituent adopt equatorial positions and relative *cis* orientation, whereas the Cl substituent itself and the methyl group adjacent to the amide have axial orientations relative to the mean plane of the five-membered ring. The conformation of the mol­ecule is stabilized by one classical N—H⋯O (2.18 Å) and one non-classical C—H⋯O (2.23 Å) hydrogen bond, each possessing an *S*(6) graph-set motif. The crystal packing is defined by several non-classical intra­molecular hydrogen bonds, as well as by partial stacking of the aromatic rings.

## Abstract 2

The crystal structure of the title compound allowed the *cis* substituent orientation on the cyclo­penta­none ring to be established. The mol­ecular conformation and crystal packing are governed by a network of hydrogen bonds and by π–π stacking.

## Chemical context   

The title compound, *cis*-3-chloro-1,3-dimethyl-*N*-(4-nitro­phen­yl)-2-oxo­cyclo­pentane-1-carboxamide, (1), was prepared in the course of study of the formation and reactivity of methacryloyl chloride dimers (2), (3) and (4) (Warneke *et al.*, 2014 ▶). The scheme below shows the reactivity of methacryloyl dimers and the synthesis of the title compound (1) (LA = Lewis acid). 
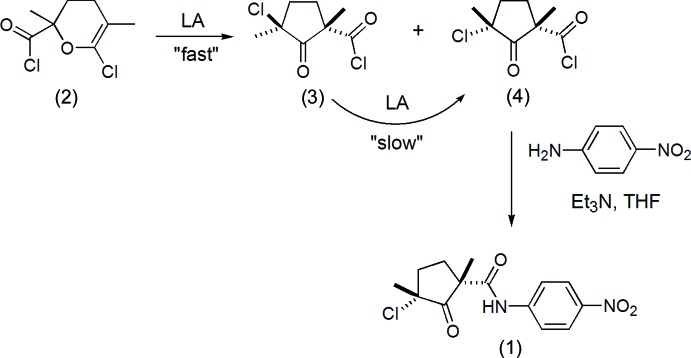


Dimer (2) forms in the oxa-Diels–Alder reaction of two methacryloyl chloride mol­ecules and, in the presence of a Lewis acid (LA, such as AlCl_3_ or TiCl_4_), rearranges to cyclo­penta­none derivatives (3) (kinetic product) and (4) (thermodynamic product). Compounds (3) and (4) show similar ^1^H and ^13^C NMR spectra, making the direct assignment of the relative orientation of the cyclo­penta­none substituents almost impossible. The crystal structure of (1), as well as the crystal structure of another aromatic amide, *cis*-3-chloro-*N*-(3,5-dichloro­phen­yl)-1,3-dimethyl-2-oxo­cyclo­penta­necarboxamide, solved and reported earlier (Warneke *et al.*, 2014 ▶), were crucial for the determination of the substituent orientation of the cyclo­penta­none ring after the isolation and derivatization of (4). For the X-ray structures of related *trans*-3-chloro-*N*-(3,5-di­chloro­phen­yl)-1,3-dimethyl-2-oxo­cyclo­penta­ne­carboxamide with *cis* orientation of two methyl groups, see Fischer *et al.* (1985 ▶).

## Structural commentary   

**Figure 1:**
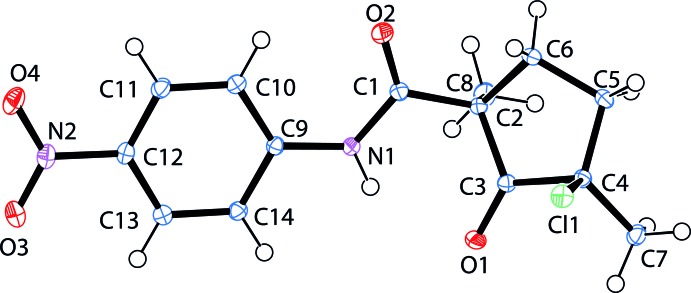
Plot of the title mol­ecule, (1), with the atom-numbering scheme. Displacement ellipsoids are represented at 50% probability levels.

**Figure 2:**
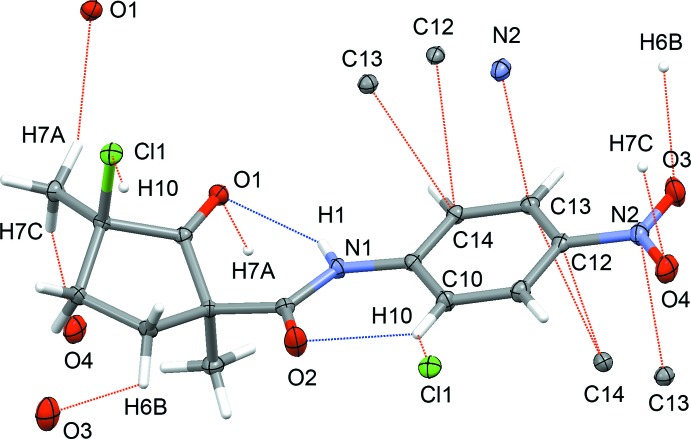
Plot of compound (1) depicting one classical N1—H1⋯O1 and one non-classical C10—H10⋯O2 intra­molecular hydrogen bond (blue), as well as inter­molecular inter­actions with distances shorter than van der Waals contacts (red).

The mol­ecular structure of the title compound with atom numbering is shown in Fig. 1 ▶. All bond lengths and angles may be considered normal. The crystal structure shows the *cis* disposition of the two methyl substituents of the cyclo­pentan­one ring. The C1 and C7 substituents adopt equatorial, whereas the C8 and Cl1 substituents have axial orientations relative to the mean plane of the five-membered ring. The 4-nitro­anilide group is essentially planar, with a maximum deviation of fitted atoms from the least-square plane, which is defined by atoms C9–C14, N1, N2, O1 and O2, of 0.0139 (9) Å for N1. The conformation of the amide is stabilized by one classical N1—H1⋯O1 (2.18 Å) and one non-classical C10—H10⋯O2 (2.23 Å) hydrogen bonds (Fig. 2 ▶), both with an *S*(6) graph-set motif (Bernstein *et al.*, 1995 ▶).

## Supra­molecular features   

**Figure 3:**
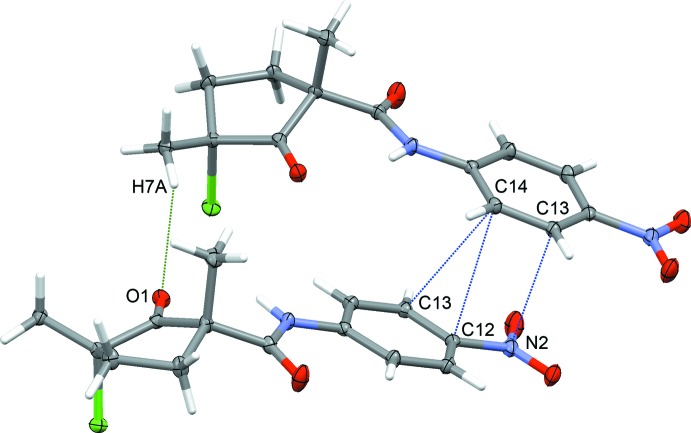
Plot of the pair of enanti­omeric mol­ecules of (1), showing short contacts between two aromatic rings and the C7—H7*A*⋯O1 hydrogen bond.

**Figure 4:**
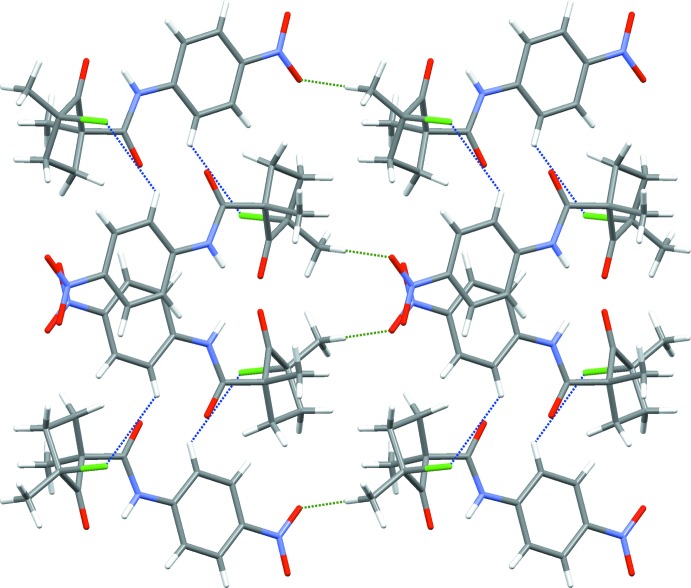
Crystal packing of (1), viewed along the *c* axis. C10—H10⋯Cl1 contacts are shown as blue dashed lines and C7—H7*C*⋯O4 contacts as green dashed lines.

The crystal packing is governed by several short contacts, which may be classified as non-classical hydrogen bonds (for reviews on weak non-classical hydrogen bonding, see Desiraju & Steiner, 1999 ▶; Steiner, 2002 ▶; Desiraju, 2005 ▶), and by partial stacking of the aromatic rings. Mol­ecules of the title compound form columns with alternating enanti­omeric mol­ecules along the *c* axis. Although no tight stacking of the aromatic rings can be established [distance between the ring centroids of 4.3719 (6) Å], the aromatic rings of neighboring mol­ecules show partial stacking with several short contacts centered near their nitro-substituent: C14⋯C13^i^ [3.3843 (15) Å; symmetry code: (i) *x*, −*y* + 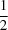, *z* + 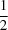], C14⋯C12^i^ [3.2483 (15) Å], and C13⋯N2^i^ [3.1860 (14) Å]. The C7—H7*A*⋯O1^i^ hydrogen bond (2.53 Å) provides additional cohesion between neighboring enanti­omeric mol­ecules in the columns (Table 1 ▶; Fig. 3 ▶). Along the *b* axis, parallel columns are inter­connected by C10—H10⋯Cl1^iii^ [2.86 Å; symmetry code: (iii) −*x* + 1, −*y* + 1, −*z* + 1], and along the *a* axis by C7—H7*C*⋯O4^ii^ [2.54 Å; symmetry code: (ii) *x* + 1, *y*, *z* + 1] non-classical hydrogen bonds (Fig. 4 ▶). Although the C6—H6*B*⋯O3^v^ [2.68 Å; symmetry code: (v) −*x* + 1, *y* + 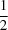, −*z* + 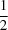] contact also lies below the sum of van der Waals radii, its classification as a hydrogen bond is disputable due to an unfavorable angle of 108°.

## Synthesis and crystallization   

The title compound was prepared as described by Warneke *et al.* (2014 ▶) by reaction of 4-nitro­aniline and *cis*-3-chloro-1,3-dimethyl-2-oxo­cyclo­penta­necarbonyl chloride in the presence of Et_3_N in THF. The product was purified by column chromatography on SiO_2_ (CHCl_3_) and readily afforded large transparent X-ray quality crystals upon slow evaporation of CHCl_3_/heptane solution (m.p. 402–403 K). ^1^H NMR (360 MHz, CDCl_3_): δ 8.89 (*bs*, 1H), 8.26–8.16 (*m*, 2H), 7.78–7.70 (*m*, 2H), 2.91–2.78 (*m*, 1H), 2.49–2.40 (*m*, 1H), 2.12–2.05 (*m*, 1H), 2.05–1.98 (*m*, 1H), 1.75 (*s*, 3H), 1.51 (*s*, 3H). ^13^C NMR (90 MHz, CDCl_3_): δ 212.4, 168.9, 143.7, 143.3, 125.0, 119.3, 69.7, 55.0, 35.6, 29.4, 25.0, 24.1. MS (EI): *m*/*z* (%) 310 (85) [*M*]^+.^, 173 (85) [*M*–NHAr]^+^. HRMS (EI): *m*/*z* [*M*]^+^ calculated for C_14_H_15_ClN_2_O_4_ 310.07203, found 310.07170.

## Refinement   

Crystal data, data collection and structure refinement details are summarized in Table 2 ▶. H atoms were included at calculated positions using a riding model, with aromatic, methyl and amide C—H bond lengths of 0.99, 098 and 0.95 Å, respectively, and amide N—H bond lengths of 0.88 Å. The *U*_iso_(H) values were fixed at 1.5*U*_eq_(C) for methyl H atoms, and 1.2*U*_eq_(C,N) for all other carrier atoms.

## Supplementary Material

Crystal structure: contains datablock(s) 1. DOI: 10.1107/S1600536814017711/hg5403sup1.cif

Structure factors: contains datablock(s) 1. DOI: 10.1107/S1600536814017711/hg54031sup2.hkl

CCDC reference: 1017486

Additional supporting information:  crystallographic information; 3D view; checkCIF report

## Figures and Tables

**Table 1:** Hydrogen-bond geometry (Å, °)

*D*—H⋯*A*	*D*—H	H⋯*A*	*D*⋯*A*	*D*—H⋯*A*
N1—H1⋯O1	0.88	2.18	2.8536 (12)	134
C10—H10⋯O2	0.95	2.23	2.8467 (14)	122
C7—H7*A*⋯O1^i^	0.98	2.53	3.3577 (13)	142
C7—H7*C*⋯O4^ii^	0.98	2.54	3.4898 (15)	165
C10—H10⋯Cl1^iii^	0.95	2.86	3.5362 (10)	129
C14—H14⋯Cl1^iv^	0.95	2.96	3.9034 (10)	171
C6—H6*B*⋯O3^v^	0.99	2.68	3.1440 (14)	109

Symmetry codes: (i) 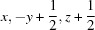; (ii) 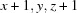; (iii) 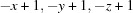; (iv) 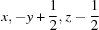; (v) 
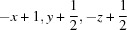
.

**Table 2:** Experimental details

Crystal data	
Chemical formula	C_14_H_15_ClN_2_O_4_
*M*_r_	310.73
Crystal system, space group	Monoclinic, *P*2_1_/*c*
Temperature (K)	100
*a*, *b*, *c* (Å)	11.4117 (4), 16.1679 (7), 7.8201 (3)
β (°)	103.382 (2)
*V* (Å^3^)	1403.66 (10)
*Z*	4
Radiation type	Mo *K*α
μ (mm^−1^)	0.29
Crystal size (mm)	0.28 × 0.18 × 0.16

Data collection	
Diffractometer	Bruker D8 Quest CMOS
Absorption correction	Multi-scan (*SADABS*; Bruker, 2014 ▶)
*T*_min_, *T*_max_	0.681, 0.747
No. of measured, independent and observed [*I* > 2σ(*I*)] reflections	15441, 6627, 5116
*R*_int_	0.028
(sin θ/λ)_max_ (Å^−1^)	0.862

Refinement	
*R*[*F* ^2^ > 2σ(*F* ^2^)], *wR*(*F* ^2^), *S*	0.046, 0.114, 1.06
No. of reflections	6627
No. of parameters	192
H-atom treatment	H-atom parameters constrained
Δρ_max_, Δρ_min_ (e Å^−3^)	0.58, −0.36

Computer programs: *APEX2* and *SAINT* (Bruker, 2014 ▶), *SHELXS97* and *SHELXL2013* (Sheldrick, 2008 ▶), *SHELXLE* (Hübschle *et al.*, 2011 ▶), *ORTEP-3 for Windows* (Farrugia, 2012 ▶), *Mercury* (Macrae *et al.*, 2006 ▶), *publCIF* (Westrip, 2010 ▶) and *enCIFer* (Allen *et al.*, 2004 ▶).

## supplementary crystallographic information

### Crystal data

**Table:**

C_14_H_15_ClN_2_O_4_	*D*_x_ = 1.470 Mg m^−^^3^
*M**_r_* = 310.73	Melting point: 402 K
Monoclinic, *P*2_1_/*c*	Mo *K*α radiation, λ = 0.71073 Å
*a* = 11.4117 (4) Å	Cell parameters from 8547 reflections
*b* = 16.1679 (7) Å	θ = 2.5–37.8°
*c* = 7.8201 (3) Å	µ = 0.29 mm^−^^1^
β = 103.382 (2)°	*T* = 100 K
*V* = 1403.66 (10) Å^3^	Block, colourless
*Z* = 4	0.28 × 0.18 × 0.16 mm
*F*(000) = 648	

### Data collection

**Table:**

Bruker D8 Quest CMOS diffractometer	6627 independent reflections
Radiation source: I-mu-S microsource X-ray tube	5116 reflections with *I* > 2σ(*I*)
'laterally graded multilayer (Goebel) mirror' monochromator	*R*_int_ = 0.028
ω and phi scans	θ_max_ = 37.8°, θ_min_ = 2.2°
Absorption correction: multi-scan (*SADABS*; Bruker, 2014)	*h* = −19→17
*T*_min_ = 0.681, *T*_max_ = 0.747	*k* = −21→27
15441 measured reflections	*l* = −12→9

### Refinement

**Table:**

Refinement on *F*^2^	Primary atom site location: structure-invariant direct methods
Least-squares matrix: full	Secondary atom site location: difference Fourier map
*R*[*F*^2^ > 2σ(*F*^2^)] = 0.046	Hydrogen site location: inferred from neighbouring sites
*wR*(*F*^2^) = 0.114	H-atom parameters constrained
*S* = 1.06	*w* = 1/[σ^2^(*F*_o_^2^) + (0.0482*P*)^2^ + 0.5524*P*] where *P* = (*F*_o_^2^ + 2*F*_c_^2^)/3
6627 reflections	(Δ/σ)_max_ = 0.001
192 parameters	Δρ_max_ = 0.58 e Å^−^^3^
0 restraints	Δρ_min_ = −0.36 e Å^−^^3^

### Special details

**Table:**

Geometry. All e.s.d.'s (except the e.s.d. in the dihedral angle between two l.s. planes) are estimated using the full covariance matrix. The cell e.s.d.'s are taken into account individually in the estimation of e.s.d.'s in distances, angles and torsion angles; correlations between e.s.d.'s in cell parameters are only used when they are defined by crystal symmetry. An approximate (isotropic) treatment of cell e.s.d.'s is used for estimating e.s.d.'s involving l.s. planes.

### Fractional atomic coordinates and isotropic or equivalent isotropic displacement parameters (Å^2^)

**Table:**

	*x*	*y*	*z*	*U*_iso_*/*U*_eq_	
C1	0.64522 (9)	0.42645 (6)	0.37961 (14)	0.01331 (17)	
C2	0.76549 (9)	0.42549 (6)	0.51672 (14)	0.01232 (17)	
C3	0.77769 (9)	0.35857 (6)	0.65852 (13)	0.01139 (16)	
C4	0.83111 (9)	0.39694 (6)	0.83990 (13)	0.01175 (16)	
C5	0.87160 (10)	0.48290 (6)	0.79734 (15)	0.01608 (19)	
H5A	0.9561	0.4818	0.7857	0.019*	
H5B	0.8649	0.5228	0.8906	0.019*	
C6	0.78602 (10)	0.50660 (6)	0.62191 (14)	0.01600 (19)	
H6A	0.7091	0.5285	0.6412	0.019*	
H6B	0.8231	0.5489	0.5596	0.019*	
C7	0.92411 (9)	0.34331 (6)	0.96019 (14)	0.01468 (18)	
H7A	0.8894	0.2888	0.9723	0.022*	
H7B	0.9483	0.3695	1.0760	0.022*	
H7C	0.9947	0.3370	0.9100	0.022*	
C8	0.86666 (10)	0.40959 (7)	0.41801 (16)	0.0181 (2)	
H8A	0.8532	0.3561	0.3578	0.027*	
H8B	0.9449	0.4091	0.5024	0.027*	
H8C	0.8658	0.4536	0.3315	0.027*	
C9	0.50005 (9)	0.33261 (6)	0.18533 (13)	0.01140 (16)	
C10	0.43082 (10)	0.39401 (6)	0.08226 (14)	0.01465 (18)	
H10	0.4525	0.4506	0.1006	0.018*	
C11	0.33034 (9)	0.37168 (6)	−0.04677 (14)	0.01470 (18)	
H11	0.2830	0.4129	−0.1173	0.018*	
C12	0.29953 (9)	0.28902 (6)	−0.07204 (14)	0.01272 (17)	
C13	0.36802 (9)	0.22669 (6)	0.02663 (14)	0.01327 (17)	
H13	0.3463	0.1702	0.0063	0.016*	
C14	0.46849 (9)	0.24892 (6)	0.15510 (14)	0.01253 (17)	
H14	0.5165	0.2073	0.2233	0.015*	
N1	0.60174 (8)	0.35022 (5)	0.31966 (12)	0.01282 (15)	
H1	0.6424	0.3073	0.3715	0.015*	
N2	0.19374 (8)	0.26654 (6)	−0.20886 (13)	0.01633 (17)	
O1	0.75274 (7)	0.28588 (5)	0.63432 (10)	0.01533 (15)	
O2	0.59793 (8)	0.49203 (5)	0.32621 (13)	0.02455 (19)	
O3	0.16813 (9)	0.19301 (6)	−0.23155 (13)	0.0270 (2)	
O4	0.13478 (8)	0.32247 (6)	−0.29704 (12)	0.02274 (18)	
Cl1	0.69827 (2)	0.40657 (2)	0.93120 (4)	0.01568 (6)	

### Atomic displacement parameters (Å^2^)

**Table:**

	*U*^11^	*U*^22^	*U*^33^	*U*^12^	*U*^13^	*U*^23^
C1	0.0149 (4)	0.0129 (4)	0.0118 (4)	−0.0017 (3)	0.0024 (4)	0.0009 (3)
C2	0.0143 (4)	0.0112 (4)	0.0107 (4)	−0.0023 (3)	0.0015 (3)	0.0011 (3)
C3	0.0117 (4)	0.0114 (4)	0.0113 (4)	0.0001 (3)	0.0031 (3)	0.0001 (3)
C4	0.0125 (4)	0.0121 (4)	0.0106 (4)	−0.0012 (3)	0.0026 (3)	−0.0003 (3)
C5	0.0191 (5)	0.0129 (4)	0.0147 (5)	−0.0057 (3)	0.0008 (4)	−0.0001 (3)
C6	0.0217 (5)	0.0106 (4)	0.0140 (5)	−0.0040 (3)	0.0007 (4)	0.0010 (3)
C7	0.0123 (4)	0.0166 (4)	0.0143 (5)	0.0007 (3)	0.0013 (4)	0.0018 (3)
C8	0.0159 (5)	0.0241 (5)	0.0151 (5)	−0.0027 (4)	0.0049 (4)	0.0018 (4)
C9	0.0112 (4)	0.0124 (4)	0.0106 (4)	0.0001 (3)	0.0025 (3)	0.0002 (3)
C10	0.0147 (4)	0.0129 (4)	0.0150 (5)	0.0009 (3)	0.0007 (4)	0.0015 (3)
C11	0.0133 (4)	0.0154 (4)	0.0145 (5)	0.0019 (3)	0.0014 (4)	0.0019 (3)
C12	0.0102 (4)	0.0174 (4)	0.0108 (4)	0.0005 (3)	0.0028 (3)	−0.0004 (3)
C13	0.0131 (4)	0.0137 (4)	0.0132 (4)	−0.0002 (3)	0.0034 (4)	−0.0004 (3)
C14	0.0126 (4)	0.0124 (4)	0.0124 (4)	0.0009 (3)	0.0025 (3)	0.0004 (3)
N1	0.0128 (4)	0.0109 (3)	0.0131 (4)	0.0001 (3)	−0.0003 (3)	0.0006 (3)
N2	0.0128 (4)	0.0224 (4)	0.0131 (4)	−0.0007 (3)	0.0016 (3)	−0.0007 (3)
O1	0.0203 (4)	0.0104 (3)	0.0146 (4)	−0.0009 (2)	0.0026 (3)	−0.0009 (2)
O2	0.0275 (4)	0.0131 (3)	0.0261 (5)	0.0012 (3)	−0.0081 (4)	0.0022 (3)
O3	0.0256 (4)	0.0228 (4)	0.0265 (5)	−0.0073 (3)	−0.0064 (4)	−0.0012 (3)
O4	0.0163 (4)	0.0281 (4)	0.0204 (4)	0.0043 (3)	−0.0025 (3)	0.0038 (3)
Cl1	0.01539 (11)	0.01668 (11)	0.01600 (12)	0.00173 (8)	0.00574 (9)	−0.00161 (8)

### Geometric parameters (Å, º)

**Table:**

C1—O2	1.2189 (13)	C8—H8A	0.9800
C1—N1	1.3701 (13)	C8—H8B	0.9800
C1—C2	1.5338 (15)	C8—H8C	0.9800
C2—C3	1.5324 (14)	C9—C10	1.4017 (14)
C2—C6	1.5369 (14)	C9—N1	1.4025 (13)
C2—C8	1.5515 (15)	C9—C14	1.4061 (13)
C3—O1	1.2136 (12)	C10—C11	1.3884 (15)
C3—C4	1.5390 (14)	C10—H10	0.9500
C4—C7	1.5169 (14)	C11—C12	1.3844 (15)
C4—C5	1.5253 (14)	C11—H11	0.9500
C4—Cl1	1.8257 (10)	C12—C13	1.3938 (14)
C5—C6	1.5371 (15)	C12—N2	1.4616 (14)
C5—H5A	0.9900	C13—C14	1.3856 (14)
C5—H5B	0.9900	C13—H13	0.9500
C6—H6A	0.9900	C14—H14	0.9500
C6—H6B	0.9900	N1—H1	0.8800
C7—H7A	0.9800	N2—O3	1.2273 (13)
C7—H7B	0.9800	N2—O4	1.2373 (13)
C7—H7C	0.9800		
			
O2—C1—N1	124.66 (10)	C4—C7—H7C	109.5
O2—C1—C2	120.13 (9)	H7A—C7—H7C	109.5
N1—C1—C2	115.13 (8)	H7B—C7—H7C	109.5
C3—C2—C1	115.45 (8)	C2—C8—H8A	109.5
C3—C2—C6	103.77 (8)	C2—C8—H8B	109.5
C1—C2—C6	111.49 (8)	H8A—C8—H8B	109.5
C3—C2—C8	106.84 (8)	C2—C8—H8C	109.5
C1—C2—C8	107.60 (9)	H8A—C8—H8C	109.5
C6—C2—C8	111.64 (9)	H8B—C8—H8C	109.5
O1—C3—C2	126.31 (9)	C10—C9—N1	123.06 (9)
O1—C3—C4	124.24 (9)	C10—C9—C14	119.77 (9)
C2—C3—C4	109.42 (8)	N1—C9—C14	117.17 (9)
C7—C4—C5	116.90 (9)	C11—C10—C9	119.64 (9)
C7—C4—C3	114.34 (8)	C11—C10—H10	120.2
C5—C4—C3	103.98 (8)	C9—C10—H10	120.2
C7—C4—Cl1	109.35 (7)	C12—C11—C10	119.64 (9)
C5—C4—Cl1	109.18 (7)	C12—C11—H11	120.2
C3—C4—Cl1	101.95 (7)	C10—C11—H11	120.2
C4—C5—C6	105.06 (8)	C11—C12—C13	121.84 (10)
C4—C5—H5A	110.7	C11—C12—N2	118.95 (9)
C6—C5—H5A	110.7	C13—C12—N2	119.20 (9)
C4—C5—H5B	110.7	C14—C13—C12	118.54 (9)
C6—C5—H5B	110.7	C14—C13—H13	120.7
H5A—C5—H5B	108.8	C12—C13—H13	120.7
C2—C6—C5	104.58 (8)	C13—C14—C9	120.55 (9)
C2—C6—H6A	110.8	C13—C14—H14	119.7
C5—C6—H6A	110.8	C9—C14—H14	119.7
C2—C6—H6B	110.8	C1—N1—C9	127.61 (9)
C5—C6—H6B	110.8	C1—N1—H1	116.2
H6A—C6—H6B	108.9	C9—N1—H1	116.2
C4—C7—H7A	109.5	O3—N2—O4	123.15 (10)
C4—C7—H7B	109.5	O3—N2—C12	118.38 (9)
H7A—C7—H7B	109.5	O4—N2—C12	118.47 (9)
			
O2—C1—C2—C3	140.81 (11)	C1—C2—C6—C5	154.86 (9)
N1—C1—C2—C3	−42.27 (13)	C8—C2—C6—C5	−84.75 (10)
O2—C1—C2—C6	22.73 (14)	C4—C5—C6—C2	−37.41 (11)
N1—C1—C2—C6	−160.35 (9)	N1—C9—C10—C11	−179.12 (10)
O2—C1—C2—C8	−100.01 (12)	C14—C9—C10—C11	1.15 (16)
N1—C1—C2—C8	76.91 (11)	C9—C10—C11—C12	0.10 (16)
C1—C2—C3—O1	47.32 (14)	C10—C11—C12—C13	−1.21 (16)
C6—C2—C3—O1	169.62 (10)	C10—C11—C12—N2	−179.78 (10)
C8—C2—C3—O1	−72.28 (13)	C11—C12—C13—C14	1.02 (16)
C1—C2—C3—C4	−134.50 (9)	N2—C12—C13—C14	179.59 (9)
C6—C2—C3—C4	−12.19 (11)	C12—C13—C14—C9	0.26 (15)
C8—C2—C3—C4	105.90 (9)	C10—C9—C14—C13	−1.34 (15)
O1—C3—C4—C7	39.21 (14)	N1—C9—C14—C13	178.91 (9)
C2—C3—C4—C7	−139.02 (9)	O2—C1—N1—C9	2.35 (18)
O1—C3—C4—C5	167.84 (10)	C2—C1—N1—C9	−174.41 (10)
C2—C3—C4—C5	−10.39 (11)	C10—C9—N1—C1	4.49 (17)
O1—C3—C4—Cl1	−78.65 (11)	C14—C9—N1—C1	−175.77 (10)
C2—C3—C4—Cl1	103.11 (8)	C11—C12—N2—O3	179.12 (11)
C7—C4—C5—C6	156.09 (9)	C13—C12—N2—O3	0.51 (15)
C3—C4—C5—C6	29.04 (11)	C11—C12—N2—O4	−0.39 (15)
Cl1—C4—C5—C6	−79.18 (9)	C13—C12—N2—O4	−179.00 (10)
C3—C2—C6—C5	29.97 (11)		

### Hydrogen-bond geometry (Å, º)

**Table:**

*D*—H···*A*	*D*—H	H···*A*	*D*···*A*	*D*—H···*A*
N1—H1···O1	0.88	2.18	2.8536 (12)	134
C10—H10···O2	0.95	2.23	2.8467 (14)	122
C7—H7*A*···O1^i^	0.98	2.53	3.3577 (13)	142
C7—H7*C*···O4^ii^	0.98	2.54	3.4898 (15)	165
C10—H10···Cl1^iii^	0.95	2.86	3.5362 (10)	129
C14—H14···Cl1^iv^	0.95	2.96	3.9034 (10)	171
C6—H6*B*···O3^v^	0.99	2.68	3.1440 (14)	109

Symmetry codes: (i) *x*, −*y*+1/2, *z*+1/2; (ii) *x*+1, *y*, *z*+1; (iii) −*x*+1, −*y*+1, −*z*+1; (iv) *x*, −*y*+1/2, *z*−1/2; (v) −*x*+1, *y*+1/2, −*z*+1/2.
